# Managing Type 1 Diabetes in Neurodiverse Children and Young People: A Qualitative Exploration of Complexity and Care Challenges

**DOI:** 10.1111/dom.71363

**Published:** 2026-09-15

**Authors:** Zoe Tattersall, Sze May Ng

**Affiliations:** ^1^ Paediatric Department Mersey and West Lancashire Teaching Hospitals Ormskirk UK; ^2^ Faculty of Health, Social Care and Medicine Edge Hill University Ormskirk UK

**Keywords:** ADHD, autism, caregivers, children and young people, lived experience, neurodivergence, qualitative, Type 1 diabetes

## Abstract

**Background:**

Neurodiversity is an umbrella term encompassing multiple diagnoses including attention‐deficit/hyperactivity disorder (ADHD) and autistic spectrum conditions. Current research is limited but highlights the complexities faced by caregivers who often face unique challenges in managing Type 1 diabetes (T1D). Little is known within the literature of the experiences of children and young people (CYP) and their caregivers where both diagnoses co‐exist and how these translate to their day‐to‐day T1D management. This study aims to understand the perceived burdens of neurodiversity for CYP and their caregivers in managing their T1D.

**Methods:**

A qualitative methodology of semi‐structured in‐depth interviewing was used to explore the barriers, challenges, perceptions and impact on quality‐of‐life managing diabetes with neurodiversity. A thematic analysis was used to analyse the data.

**Results:**

Fifteen CYP (age range 12–18 years) and 13 parents caregivers were interviewed. All CYP had a diagnosis of autism, ADHD or both. Common themes identified were related to caregivers needing to adapt routines to meet individual needs. Family support was central to diabetes management. Sleep disturbance related to fear of hypoglycaemia was common, but continuous glucose monitoring improved sleep quality and reduced anxiety. Diabetes technologies, were widely perceived to reduce burden, enhance confidence and support self‐management, despite occasional sensory challenges. Caregivers expressed concerns about limited tailored resources. CYP were worried about transition to adult services. Both CYP and caregiver experiences at diagnosis were generally negative, particular issues being change in routine, fingerpricks and hospital settings. CYP did not view neurodiversity as a barrier to self‐managing their own diabetes.

**Conclusion:**

Caregivers demonstrate resilience in managing and navigating challenges and CYP did not see their neurodiversity as a barrier to managing their T1D. Diabetes technologies were viewed as improving quality of life. Day‐to‐day coping strategies varied between CYP and caregivers. Effective T1D management in CYP with neurodiversity necessitates that healthcare providers are trained to understand and address the specific needs of neurodiversity. This includes adapting communication strategies and developing individualised care plans. Future research is needed to develop tailored interventions and support systems specifically for CYP with T1D and neurodiversity.

## 1 Introduction

Type 1 diabetes (T1D) is a complex, lifelong condition requiring continuous self‐management, including insulin administration, frequent blood glucose monitoring, dietary regulation and adjustment for any physical activity. For children and young people (CYP), these demands occur alongside developmental, psychological and social transitions, making effective management particularly challenging. When T1D coexists with neurodiversity, an umbrella term encompassing conditions such as autism spectrum condition (ASC) and attention‐deficit/hyperactivity disorder (ADHD), these challenges can be significantly amplified. There has been a rise in CYP diagnosed with neurodivergent conditions [1, 2] and rates of neurodiversity are higher in those with T1D than in the general population [3]. We know that those with T1D and a diagnosis of a neurodiverse condition may lead to poorer glycaemic management and higher risk of complications [4]. Therefore, understanding CYP and caregivers' experiences of how these conditions co‐exist and affect one another is vital to improving outcomes for CYP with neurodiversity.

Neurodiverse individuals often experience differences in communication, executive functioning, sensory processing and emotional regulation [5]. These differences can directly impact diabetes self‐management tasks. Current research is limited but highlights the complexities faced by caregivers who often face unique challenges in managing T1D. Little is known within the literature of the experiences of CYP and their families where both diagnoses co‐exist and how these translate to their day‐to‐day T1D management.

Caregivers frequently report heightened stress, increased caregiving burden and challenges navigating healthcare systems that are not always tailored to neurodiverse needs [6]. However, despite growing recognition of these challenges, there is limited qualitative research capturing both child and caregiver perspectives on managing T1D within the context of neurodiversity. Understanding these lived experiences is crucial to developing effective, person‐centred care models. Furthermore, with increasing use of diabetes technologies such as insulin pumps, hybrid closed loop systems and continuous glucose monitoring (CGM), it is important to explore how these innovations impact this specific population. This study aims to address this gap by exploring the perceptions, challenges and how neurodivergence influences the day‐to‐day management of T1D in CYP with neurodiversity, the experiences of caregivers and to identify the adaptations and support needs reported by young people and their caregivers. By identifying key themes, it seeks to inform the future development of tailored, solution‐focused frameworks within diabetes services.

## 2 Method

A qualitative design was adopted to enable an in‐depth exploration of participants' experiences. Semi‐structured interviews were conducted with CYP who had a diagnosis of T1D and coexisting neurodiversity and their primary caregivers. Participants were recruited from a paediatric diabetes service within a large UK teaching hospital using a purposive sampling approach to capture a range of experiences. Interviews were conducted either face‐to‐face or via a secure virtual‐telemedicine platform. All interviews were audio‐recorded and transcribed verbatim. An inductive thematic approach was used, beginning with repeated familiarisation with the transcripts, followed by systematic coding of meaningful segments of data. Related codes were grouped into candidate themes, which were iteratively reviewed, discussed and refined by the team against the original transcripts. Interviews were conducted by a paediatrician with more than 12 years of post‐medical qualification clinical experience, who received supervision and guidance in qualitative interviewing from a qualitative researcher. The interviewer had no pre‐existing clinical relationship with the participants. Interviews were arranged flexibly according to participant preference and were undertaken either face‐to‐face in the paediatric diabetes clinic or remotely using a secure telemedicine platform. The semi‐structured interview guide was developed from the aims, relevant literature and experience of caring for CYP with both T1D and neurodiversity. Questions were designed to explore key areas including day‐to‐day diabetes management, sensory experiences, routines, technology, family support, interactions with healthcare professionals and transition. The guide was used flexibly rather than as a rigid questionnaire, with wording, pace, prompting and the order of questions adapted according to the participant's communication preferences, level of understanding and responses. Additional processing time, clarification, repetition and caregiver support were used where appropriate.

The project was formally registered as a service evaluation audit (MWL No. 23‐353). The classification was made through the Trust's Audit department responsible for service‐evaluation audit governance. Participation was entirely voluntary from both CYP and caregiver. Written informed consent was obtained for all participants. The consent process included permission for interviews to be audio‐recorded, transcribed and analysed and for anonymised excerpts from interviews to be reproduced in resulting publications and presentations. Participants were informed that participation was voluntary, that their responses would remain confidential and that they could withdraw in accordance with the arrangements described in the participant information provided. Given the neurodiverse population, interviews were conducted flexibly and communication was adapted to participants' individual needs. This included allowing additional processing time, using clear and direct language and offering interviews either face‐to‐face or through a secure virtual telemedicine platform. Safeguarding procedures followed existing policies any disclosure raising a concern regarding the CYP's welfare would have been managed through established Trust safeguarding pathways. No identifiable information is included in the quotations reported in the manuscript. Participants were assured of confidentiality and their right to withdraw at any stage.

## 3 Results

Fifteen CYP and 13 caregivers were interviewed. Demographics and diagnoses for CYP are shown below (Table 1). Neurodevelopmental diagnoses were established from participants' clinical records and reflected formal diagnoses previously made by the appropriate clinical services. Patient ages ranged from 12 to 18 years, mean age of CYP was 15.13 years (SD = 3.04) and mean duration of diabetes was 4.9 years (SD = 3.53). All participants were wearing a CGM sensor and were on a hybrid‐closed loop system.

**TABLE 1:** Demographics for children and young persons.

Age (years)	15.13 ± 3.0 SD
Age at diagnosis (years)	10.33 ± 2.1 SD
Duration of diabetes	4.9 ± 3.5 SD
Gender	
Male	11
Female	4
Ethnicity	
White British	14
Other	1
Diagnosis	
ASD	7
ADHD	4
ASD + ADHD	4

Areas of discussion that represent the opinions of the young person (YP) and their parents/carer giver (CG) are identified with included quotes throughout the different themes to help to illustrate perspectives transparently.

### 3.1 Family Support

A central theme that emerged from the data was the importance of support structures within the family (Table 2). Caregivers described playing an active and ongoing role in diabetes management, often tailoring their approaches to accommodate their child's specific needs (Quote 1). A frequently occurring theme from CYP was ‘learning to deal with it’ or ‘getting on with it’ (Quote 2). Caregivers also discussed their feelings of responsibility and it being their ‘job’. Caregivers often relied on extended families to support with diabetes management. They also commented on the significant impact that a diagnosis of diabetes and neurodiversity has on the rest of the family including siblings (Quote 3). Structured routines were frequently reported as beneficial, although disruptions to routine could present challenges. Participants discussed feeling anxious about changes (Quote 4). Caregivers use reminders, rewards and visuals to help with diabetes management (Quotes 5 and 6). One YP explained things worked better if he was in control and applied the pump themselves. CYP often reported that it was the fear of the unknown that caused the greatest issue (Quote 7). They also reported incidents of having to change treatments in order to reduce sensory challenges they faced. Even when better and newer diabetes technology emerged, CYP were keen to stick with what they already knew and disliked change (Quote 8).

**TABLE 2:** Family support.

Quote no.	Quotes
1.	I think that she's got plenty of time in life to be handling that herself. So she needs to enjoy her childhood. So what we've done is we've taken complete control of her diabetes. (CG)
2.	I just kind of learned to deal with it, over the years really. (YP)
3.	It doesn't just affect our family home, it affects the whole family I feel. (CG)
4.	I had to adapt to the needles and cannulas. I had to get used to them as it makes me anxious. You count down don't you, I think routine definitely helps. (YP)
5.	He thrives off those kind of reward charts and stickers and things. (CG)
6.	We do hold hands when his pod is going in and we use lot of visuals. (CG)
7.	I think it's the not knowing again, because even getting this sensor on, it was gonna hurt, but then now it's not as bad as you thought it is. (YP)
8.	Why would I change if I know it works? I don't like change. (YP)

### 3.2 Technology

The introduction of CGM technology was widely perceived as transformative, providing reassurance through real‐time glucose data and alarms. Both caregivers and CYP reported improvements in sleep quality and a reduction in anxiety (Table 3). They also consistently reported that insulin pumps and CGM systems reduced the burden of diabetes care, enhanced confidence and provided a sense of freedom (Quotes 1 and 2). Many CYP and caregivers were frustrated by the frequency of alarms and reported keeping alarms on quiet due to overstimulation and anxiety (Quotes 3 and 4). Challenges related to sensory sensitivities, particularly with device wear, were noted, although participants commented that overall, this was better than fingerpicking and giving insulin via pens (Quotes 5 and 6).

**TABLE 3:** Technology.

Quote no.	Quotes
1.	It wasn't until we're on the Dexcom and pump with the follower app that literally changed my life that day. It literally gave me the ability to sleep again. (CG)
2.	Going from, you know, stabbing myself every time I wanted to eat to just putting in a few buttons. I mean, obviously I have to change it every couple of days, but the freedom is immense. (YP)
3.	I get over simulated dead easily and then, like my pod can be beeping constantly, and I'm like, oh, like it's too much and get dead overwhelmed. (YP)
4.	He gets embarrassed really easily when his device starts going beep beep. He is still embarrassed about that noise going off because that's about him. That it's attention on him. Do you know what I mean? … attention that he doesn't want it. (CG)
5.	He doesn't like them on his body or the sounds. He did used to silence them, but I think school he will silence it because he doesn't want everyone else to hear it. In primary school he did that. He doesn't like the sounds of the alarms. It was still better than injections of fingerpricks. (CG)
6.	As with most things, if you hear something enough, you kind of get a bit numb to it. So, there's not much you can do to fix that necessarily. I change the alarms occasionally just kind of to so it's not quite a stagnant, but like, there's nothing really you can do about that necessarily. (YP)

### 3.3 Transition

In relation to future transition, caregivers generally expressed confidence in their child's ability to develop independence over time (Table 4) (Quote 1). Both CYP and caregivers felt that the process should have been started earlier and they would have been more prepared (Quotes 2 and 3). Many participants commented on their nervousness about the transition process and the changes that this would entail, worried about new hospitals, different levels of communication and different management techniques (Quote 4).

**TABLE 4:** Transition.

Quote no.	Quotes
1.	He's matured quite a lot since the early years and has more confidence to transition. (CG)
2.	Maybe extra meetings so I am more familiar when it is needed. I feel like it might change … maybe a bit different. They might treat me differently. (YP)
3.	I think the kids team should have helped with a few more baby steps in between before actually doing that. (CG)
4.	I know what's going to happen, but it's just. I don't know, it's the thought of just being somewhere completely new. Like, I don't know the layout, for example of … the rooms and things like that. I know that's, like a little thing, but, like, still it's just not knowing, if that makes sense. (YP)

### 3.4 Diagnosis

Experiences of diagnosis had a significant impact on both CYP and caregivers, and many described this as the worst period they faced (Table 5) (Quotes 1 and 2). Many CYP described attempts at intravenous access, injections and fingerpricks being the scariest and explained that a little more time taken to explain things would have significantly improved their experiences (Quote 3). There was mixed feedback about the education that was given while an inpatient. Some felt that more information was needed immediately after diagnosis and others felt that there was an initial overload of information (Quotes 4 and 5). They also reported issues with bedding, noise and lighting given their hypersensitivities (Quotes 6 and 7).

**TABLE 5:** Diagnosis.

Quote no.	Quotes
1.	I remember being actually terrified. Saying that I want to go home, I want to go home to go home. (YP)
2.	I know it's a lot of information to take in, but I was just so confused as to what was going on. As far as I'm concerned, it's like my child's screaming the place down and someone's coming in, just jabbing … all through the night. (CG)
3.	They'd put a cannula in arm and I was like, kicking off, so I was like, I don't want a cannula, because it just scared me because I've never had one before and it was all just it was literally I felt like I was being pinned down, like mentally because I was just panicking because I was just so overwhelmed. (YP)
4.	I know that diabetes team come and do the education kind of after, but I suppose maybe start some of the basics of education when you come in at diagnosis and say why we're doing the things that we are. The initial overload, I think, is quite intense. (CG)
5.	It was the waiting for his food before insulin. He just wanted his food. He couldn't understand why he had to wait for his food. (CG)
6.	The sheets are awful and rough. The blankets are somehow too heavy and too thin. Very challenging for autistic child. The thing with him as well though at the ward, what I do remember it was the noise of all the other kids on the ward. It was too loud. I think he would have benefited of being in his own room. (CG)
7.	Obviously the lights are very bright and that's what you struggle with… You struggle with the light that's above the bed often we have to get that switched off because he doesn't like that, especially in the night when he's trying to sleep. (CG)

### 3.5 Fear of Sickness, Complications and Hypoglycaemia

Fear of sickness, complications, sleep and night‐time management were prominent concerns noted (Table 6). Caregivers described significant anxiety related to nocturnal hypoglycaemia, often leading to disrupted sleep due to frequent monitoring (Quotes 1). Fear of hypoglycaemia was a pervasive theme, influencing both emotional wellbeing and management behaviours (Quote 2). The availability of CGM data and alerts was reported to alleviate some of this fear (Quote 3). The fear of sickness was often a driving force in CYP changing pumps or sensors despite their sensory difficulties (Quotes 4 and 5). Many caregivers described being fixated on HbA1c levels and fear of complications (Quotes 6).

**TABLE 6:** Fear of sickness.

Quote no.	Quotes
1.	I didn't sleep for, I reckon, five years, probably, of just not sleeping properly. (YP)
2.	I don't wanna to go to a cinema because if I go hypo it, I realise or I've walked too much that day, I don't want to die. (YP)
3.	My Dexcom, might actually have been world changing to be honest. (YP)
4.	He knows that it is life or death. Because he knows how important it is, he is not one to not do it. (CG)
5.	The thought of if I don't do it I could not feel well. And I want to feel well and the thought of making my blood sugar's go up because I've not changed it. (YP)
6.	I am fixated on her HbA1c and she worries over death that things a child should not worry about. (CG)

### 3.6 Interactions With Healthcare Professionals

Relationships with healthcare professionals were described positively and participants valued accessible, supportive and non‐judgemental care (Table 7), In particular they highlighted the importance of continuity, consistency and trust (Quotes 1 and 2). Within the clinic setting some CYP commented on fearing loud noises and needing to use headphones and watch videos, whereas other CYP commented on the fact they did not find the waiting room an issue and felt it was always quiet. Others also raised their difficulties with telephone conversations and would prefer to communicate via email or text (Quote 3). Access to psychological support was particularly valued and so was access to school education sessions (Quote 4). Caregivers and CYP both identified a lack of structured resources specifically addressing the intersection of neurodiversity and T1D. With regards to information given at diagnosis a lack of child‐friendly written information was highlighted (Quotes 5).

**TABLE 7:** Interactions with professionals.

Quote no.	Quotes
1.	He has felt that they're his team rather than just a random doctor. And I think that makes a huge difference. (CG)
2.	I personally think that if you've got someone who's neurodivergent, that consistency is really important. Maybe with other people interspersed to show that, you know, but it might require that doctor coming in with May one time, but it to stop the caveat. Especially the younger CYP that safety is definitely important. (CG)
3.	I don't like phone calls. I hate speaking to people over the phone. I'd rather speak face to face or like emails. I prefer emails or stuff like that. I think ADHD comes with anxiety, so I think that's just like. (YP)
4.	And I do also think that the combined care with [the psychologist] has been literally a lifesaver for us that's been significant in the process. (CG)
5.	The stuff they give you to try and understand it is not kid friendly. You have to go at your way to find that kind of stuff. I guess it's not something you've given a hospital. (YP)

### 3.7 Perceptions of Neurodiversity

Notably, CYP did not perceive their neurodiversity as a barrier to managing their diabetes (Table 8) (Quotes 1 and 2). Instead, many viewed it as part of their identity, while caregivers emphasised resilience and adaptation in navigating challenges (Quote 3). Structure in routine and eating habits was a common benefit of neurodivergence with regards to diabetes management (Quote 4). Some highlighted their hyper‐focussed manners as a benefit in understanding diabetes education and felt confident in their carb‐counting skills (Quote 6).

**TABLE 8:** Perceptions of neurodiversity.

Quote no.	Quotes
1.	I'm not really sure it has had the effect of my diabetes for the most part. (YP)
2.	Not really necessarily a positive or negative effect. It just kind of does change the process of it all. (YP)
3.	I have kind of like ADHD and autism proofed life now as a mom. (CG)
4.	I think that's a positive effect more than anything because it's so structured. I think he just naturally, he likes routine like that, so it just falls into place, I think. (CG)
5.	I think it helps me learn things because. I hyper‐focused on it a little bit. (YP)
6.	It's just things have to be done a certain way. Even if it's not practical at the time, it has to be done the same way, which can be a bit of a disadvantage. I like visuals, like pictures and like diagrams and things. (YP)

Across several themes, participants described a strong preference for predictability, familiarity and control. Changes in routines, unfamiliar procedures and new technologies could increase anxiety or sensory discomfort, whereas gradual introduction, repetition and allowing the YP greater control appeared to facilitate engagement.

## 4 Discussion

This study provides important insights into the experiences of CYP with T1D and neurodiversity and their caregivers. Contrary to expectations, neurodiversity was not universally perceived as a barrier to effective diabetes management. Instead, both CYP and caregivers described a process of adaptation and resilience, supported by family involvement and healthcare provision. CYP often raised feelings about not wanting to be different to others and this is important when supporting them within school settings but also within the wider diabetes community. Routine was felt to be largely beneficial to those living with diabetes management and neurodivergence. Although changes to routines posed significant issues for CYP and could often lead to anxiety, these were overcome with reassurance and repetition of reminders from caregivers and their healthcare professionals. Experience has shown that including the CYP in the planning of changes can be beneficial as can allowing them time to become familiarised [7].

Sensory difficulties were identified as a significant challenge particularly with regards to blood glucose monitoring, insulin administration and initial management at diagnosis. CYP highlighted not fully being able to explain the reasons for their difficulties but emphasised their importance. Our findings were consistent with previous qualitative research [8] that focusses on those with autism and T1D. We demonstrated that sensory issues play a major role in the uptake of diabetes technologies but that these technologies can have significant improvements in quality of life for this population. Insulin pumps, hybrid closed loop and CGM systems were consistently associated with reduced anxiety, improved sleep and enhanced quality of life. These findings support the increasing emphasis on equitable access to diabetes technologies, particularly for individuals with additional needs [9]. Although participants commonly described diabetes technologies as reducing burden and providing perceived benefits for sleep, anxiety and quality of life, these outcomes were not objectively measured and should therefore be interpreted as participant‐reported experiences. Similarly, while many participants did not perceive neurodivergence itself as a barrier to diabetes management, they described important sensory, communication and organisational challenges that required adaptation and individualised support. This highlights a key tension in the findings: neurodivergence was not necessarily experienced as a barrier by participants, but effective diabetes care often depended on tailored accommodations and flexible care processes.

Barriers to overcome for CYP with neurodivergence include loud alarms, reluctance to change and difficulties with distraction between carb counting, administration of insulin boluses and eating a meal. Both CYP and caregivers suggested solutions to some of these barriers and these should be discussed with tech companies to improve accessibility. CYP described frustration at having to disrupt their activities to be able to manage their diabetes. Patient advocacy organisations such as Breakthrough T1D have published webpages for CYP with autism and ADHD [10, 11, 12]. These report similar difficulties and could be a useful tool for CYP. Fear of hypoglycaemia emerged as a central concern, consistent with existing literature [13]. The role of CGM in mitigating this fear underscores its value not only as a clinical tool but also as a means of improving psychological wellbeing. Fear of sickness was regularly brought up as a reason to comply with diabetes management and this fear should not be underestimated. The significance of mental health reiterates the need for psychological input and support integrated within multi‐disciplinary care. These findings have implications for diabetes self‐management support. Approaches that enhance autonomy, perceived control and predictability, while reducing sensory and communication barriers, may support engagement with diabetes technologies and other aspects of care. Such principles may be relevant across services, although individualised assessment remains essential.

Transition to adult services was an area of concern for CYP; however, caregivers were confident in their child's independence over time. A gradual approach to transition and early introduction was important to families, correlating with previous evidence of CYP with autism transitioning to adult services [14]. A more appropriate approach may be to introduce transition to conversations from an earlier age to help prepare CYP and their caregivers; this would allow for preparation without the need for any changes to clinical care. With regards to management at diagnosis, taking some time to explain to CYP the importance of IV access and remaining calm in these stressful situations was valuable to improving their experiences. We know that a high proportion of CYP are in DKA at diagnosis [15] and a larger proportion of CYP with neurodivergence have T1D [3]. ADHD is also associated with higher HbA1c and increased acute diabetes‐related complications [16]. Therefore, this opportunity at diagnosis could significantly improve the interactions between the medical team and CYP with neurodiversity, leading to greater trust for ongoing medical management of T1D. The lack of targeted resources for neurodiverse individuals with T1D represents a significant gap. There is a clear need for the development of structured guidance and interventions that address the unique challenges faced by this population.

The positive perceptions of healthcare teams reported are encouraging, reflecting the importance of accessible and person‐centred care. The positive impact of psychologist input in the team cannot be understated and highlights the importance of their inclusion with the diabetes multi‐disciplinary team. Some CYP and caregivers highlighted that they did not always see the same clinicians in clinic and felt that this would help with continuity and trust. Experience from psychologists [7] also demonstrates these concerns and having consistent healthcare professionals involved in the care of patients with neurodiversity is important. Other ways of overcoming these challenges have been suggested [7], such as photos of staff members in the waiting room.

Several limitations should be considered when interpreting these findings. Participants were recruited from a single UK centre, which may introduce selection bias and limit transferability to other populations and healthcare settings. The predominantly White British and male sample further limits representation of the wider population of young people with T1D and neurodiversity. Individuals who were unable or unwilling to participate in conventional verbal interviews may also be underrepresented, particularly those with more substantial communication needs. Although the interviewer had no pre‐existing clinical relationship with participants, clinician researcher and social‐desirability effects cannot be excluded. Where young people and caregivers were interviewed together, responses may have been influenced by the presence of the other participant. In addition, combining participants with different neurodevelopmental diagnoses may obscure condition‐specific experiences. Finally, participants' experiences of diabetes technology and clinical support may reflect local patterns of technology access and multidisciplinary care and may not be directly transferable to services with different resources or models of care. A further limitation is that autism and ADHD are distinct neurodevelopmental conditions with differing presentations and potentially different effects on diabetes self‐management. By grouping these conditions under the broader term of neurodiversity, condition‐specific experiences and mechanisms may have been obscured; therefore, the findings should not be assumed to apply uniformly across diagnostic groups.

## 5 Conclusion

These findings emphasise the importance of individualised care for CYP with neurodiversity. Healthcare professionals must recognise the diversity of needs within this group and adapt their approaches accordingly. This includes tailoring communication strategies, providing flexible support and involving families in care planning from diagnosis to transition. Families demonstrated resilience in navigating the dual challenges of T1D and neurodiversity. Diabetes technologies were consistently associated with improved quality of life. There posed significant organisational and sensory challenges that CYP and families overcame. Effective management of T1D in neurodiverse CYP requires healthcare professionals to develop a nuanced understanding of neurodiversity, including adapting management strategies and delivering individualised care. Future research should focus on developing tailored interventions and structured support systems to better meet the needs of this population.

## Funding

The authors have nothing to report.

## Conflicts of Interest

The authors declare no conflicts of interest.

## Data Availability

The data that support the findings of this study are not publicly available due to privacy reasons but are available from the corresponding author upon request.
